# Unraveling Mobile Health Exercise Interventions for Adults: Scoping Review on the Implementations and Designs of Persuasive Strategies

**DOI:** 10.2196/16282

**Published:** 2021-01-18

**Authors:** Karlijn Sporrel, Nicky Nibbeling, Shihan Wang, Dick Ettema, Monique Simons

**Affiliations:** 1 Faculty of Geosciences Utrecht University Utrecht Netherlands; 2 Department of Applied Psychology Amsterdam University of Applied Sciences Amsterdam Netherlands; 3 Institute of Informatics University of Amsterdam Amsterdam Netherlands; 4 Department of Information and Computing Sciences Utrecht University Utrecht Netherlands; 5 Social Sciences, Consumption and Healthy Lifestyles Wageningen University & Research Wageningen Netherlands

**Keywords:** mobile health, physical activity, goals, feedback, rewards, reminder systems, social support, adult

## Abstract

**Background:**

It is unclear why some physical activity (PA) mobile health (mHealth) interventions successfully promote PA whereas others do not. One possible explanation is the variety in PA mHealth interventions—not only do interventions differ in the selection of persuasive strategies but also the design and implementation of persuasive strategies can vary. However, limited studies have examined the different designs and technical implementations of strategies or explored if they indeed influenced the effectiveness of the intervention.

**Objective:**

This scoping review sets out to explore the different technical implementations and design characteristics of common and likely most effective persuasive strategies, namely, goal setting, monitoring, reminders, rewards, sharing, and social comparison. Furthermore, this review aims to explore whether previous mHealth studies examined the influence of the different design characteristics and technical operationalizations of common persuasive strategies on the effectiveness of the intervention to persuade the user to engage in PA.

**Methods:**

An unsystematic snowball and gray literature search was performed to identify the literature that evaluated the persuasive strategies in experimental trials (eg, randomized controlled trial, pre-post test). Studies were included if they targeted adults, if they were (partly) delivered by a mobile system, if they reported PA outcomes, if they used an experimental trial, and when they specifically compared the effect of different designs or implementations of persuasive strategies. The study methods, implementations, and designs of persuasive strategies, and the study results were systematically extracted from the literature by the reviewers.

**Results:**

A total of 29 experimental trials were identified. We found a heterogeneity in how the strategies are being implemented and designed. Moreover, the findings indicated that the implementation and design of the strategy has an influence on the effectiveness of the PA intervention. For instance, the effectiveness of rewarding was shown to vary between types of rewards; rewarding goal achievement seems to be more effective than rewarding each step taken. Furthermore, studies comparing different ways of goal setting suggested that assigning a goal to users might appear to be more effective than letting the user set their own goal, similar to using adaptively tailored goals as opposed to static generic goals. This study further demonstrates that only a few studies have examined the influence of different technical implementations on PA behavior.

**Conclusions:**

The different implementations and designs of persuasive strategies in mHealth interventions should be critically considered when developing such interventions and before drawing conclusions on the effectiveness of the strategy as a whole. Future efforts are needed to examine which implementations and designs are most effective to improve the translation of theory-based persuasive strategies into practical delivery forms.

## Introduction

Physical activity (PA) mobile health (mHealth) interventions, such as interventions delivered by wearable technologies, SMS messages, and mobile apps, have potential for supporting PA behavior [1-5]. Yet, while some PA mHealth interventions successfully increase PA, others do not [2,6,7], and it is unclear why this is the case. A possible explanation for this discrepancy in effectiveness is the various ways in which the persuasive strategies are being incorporated in PA mHealth interventions [8].

Persuasive strategies (or behavior change techniques [8]) are theoretically underpinned elements of interventions, such as goal setting or rewards, intended to foster a positive behavior or attitude change toward PA. Over the last decade, several taxonomies of persuasive strategies have been developed [8-12], including a taxonomy specifically for PA and dietary interventions (ie, the CALO-RE taxonomy [9]). These taxonomies allow for a clear and consistent description of interventions [13], and they have been frequently adopted for designing and evaluating interventions for behavior change [14-18].

Although the persuasive strategies from the taxonomies are commonly used to inform the study design, they do not contain a guideline to operationalize the strategies [19-21]. Consequently, the same persuasive strategy can be shaped differently in different exercise interventions [20,22]. For instance, the technical implementation of the strategy cue or prompt [9] can be delivered via a mobile phone as a text or sound message, by means of a flashing light or even as a vibration. However, it can also be delivered via an email or an actual phone call. Furthermore, the design characteristics of the message can also differ; messages can be framed differently (eg, positive or negatively framed) [23,24], and the messages can be short or long and generic or tailored [23,24]. As a result of these diverse implementations and designs, interventions might evoke different user responses, even though they use the same persuasive strategy. This renders it difficult to draw a conclusion about the effectiveness of the persuasive strategy at the theoretical level.

Several studies have argued that design characteristics influence the effectiveness of the strategy, such as the use of different social media features [25] or the content of messages [23,24]. However, the technical implementation of these strategies has received little attention so far (eg, the device used or the accessibility of the strategy). This is surprising, as technical implementation can influence user experience, usability, and intervention exposure, which, in turn, likely influences the effect of the intervention [18,26,27]. Thus far, only one review has examined whether both design characteristics and technical implementations could impact the effectiveness of digital exercise or dietary interventions on the persuasive strategy *feedback* [22]. A great variety in implementation forms of feedback was found, for instance, regarding the accessibility of feedback (continuous access or daily messages) and the form of feedback (visual or not). Moreover, the findings indicated that not all types of feedback were equally effective in changing PA behavior [22].

Schembre et al [22] limited their study to feedback; however, other strategies likely face the same diversity in design characteristics and technical implementations. Therefore, this scoping review sets out to explore the designs and implementations of other promising persuasive strategies in mHealth PA interventions for adults and explores whether previous mHealth studies examined the influence of the different design characteristics and technical operationalizations of common persuasive strategies on the effectiveness of the intervention to persuade the user to engage in PA. As analyzing all strategies is beyond the scope of this study, the analysis is limited to the most common and evidence-based strategies for PA [7,18], namely, monitoring, goal setting, reminders, rewards, and 2 social strategies (sharing and social comparison).

## Methods

### Approach

A nonsystematic literature search was performed to identify original research papers that examined the selected persuasive strategies in the context of PA mHealth interventions. A nonsystematic search was deemed appropriate because the objective of this study is to gain insights into the various operationalizations of strategies and their influence on the effectiveness of the strategy and *not* to provide a complete overview of current literature or a list of effective implementations and characteristics of the strategies. The PRISMA ScR (Preferred Reporting Items for Systematic Reviews and Meta-Analyses Extension for Scoping Reviews) criteria were used to guide the reporting of the methods and findings (Multimedia Appendix 1) [28]. The protocol for this review was not registered.

### Search Strategy and Study Selection

Most papers were identified by checking the references of (recent) reviews in the same field and in the author’s personal libraries. As the research team is multidisciplinary, papers from the fields of behavior change, computer science, and gamification were included. When interesting papers were identified (either by previous reviews or in the library of authors), extensive snowball searches were performed (ie, the references of all interesting papers were checked for other relevant papers). We extended the snowball search with additional quick searches in Google Scholar when the initial search resulted in a small number of studies for a specific operationalization of a persuasive strategy (eg, sharing information on social media). The search included terms that refer to mHealth (eg, mobile devices, PA apps), PA behavior (eg, exercise, walking, running), the persuasive strategy (eg, sharing, social media, Facebook, Twitter), and adults. These additional searches increased the number of identified papers to a limited extent.

Studies were included if they (1) were (partly) delivered by a mobile system (eg, pedometer, SMS, mobile app), (2) reported PA outcomes (self-reported or objectively measured), (3) used an experimental trial (eg, randomized controlled trial, factorial design, pretest-posttest design), (4) examined at least one of the selected persuasive strategies, and (5) described this strategy in sufficient detail. Finally, (6) studies were only included when they specifically compared the effect of different designs or implementations of persuasive strategies. Therefore, papers were excluded if they only examined the effectiveness of the intervention as a whole (with multiple strategies). Studies were also excluded if they targeted children or individuals with a chronic disease (eg, patients with cardiovascular diseases, mental disorders, etc), as these target groups may have different needs [26]. There were no restrictions on publication year, sample size, or study duration.

### Data Charting

To systematically analyze the included studies, a data charting list was developed in multiple review rounds with input from all coauthors (consistent with the guidelines for writing a scoping review [29]). The final data chart comprised 3 sections, namely (1) study characteristics, (2) technical implementations and design characteristics of persuasive strategies, and (3) study results (Multimedia Appendix 2). The study characteristics included information on the methodology of the study and other factors that can influence study outcomes (ie, characteristics of the participants and contextual factors [30]).

The *technical implementations and design characteristics* section was inspired by the Behavioral Intervention Technology (BIT) model [20]. The BIT model is grounded in 3 well-respected design models [11,31-33] and includes principles from both behavioral theories and technological features. The BIT delivery elements (subdivided into delivery systems and elements) and BIT workflow informed the *technical implementation* category (Multimedia Appendix 2). The BIT characteristics of the elements informed the *design characteristic* category. Most design characteristics were inferred from previous reviews [22-25] and theories of behavior change [33-35], as the BIT framework does not provide a detailed list of design characteristics. Furthermore, during the data extraction period, the chart was updated when new implementations or design characteristics were identified.

The final data chart section covered the *study results*. We examined whether there was a higher amount of PA compared with a control group (without the strategy) and compared with another intervention arm (with a different operationalization of the strategy, see Multimedia Appendix 2). The PA outcome measurement used was the step count of the participants, unless the paper did not measure this. In that case, the main PA outcome measurement was used instead, as described in this study. The outcomes were classified as *positive* when the PA outcome measurement of the study was significantly more effective, *neutral* when no effect was found, and *negative* when the implementation resulted in worse PA outcomes.

The general study characteristics were extracted for the study as a whole. The type of persuasive strategy and its design and technical implementation were extracted separately for each intervention arm because this differed between arms in the same study. One researcher (KS) performed the data extraction of all the included papers. To ensure that the data extraction was performed correctly for all persuasive strategies (in line with Levac et al [29]), the second reviewer (SW) performed data extraction of at least 20% of each of the persuasive strategies (22.6% on average, SD 0.9%). The interrater reliability was high (93.5% agreement).

## Results

### Overview

The search yielded 29 original research papers (85 intervention arms) [36-64]. An overview of the study characteristics can be found in Multimedia Appendix 3 [36-64]. The results of the individual persuasive strategies are presented below. For each strategy, first, a short description or definition is provided. Second, a summary of the identified implementations and designs in the intervention arms is provided (a complete overview can be found in Multimedia Appendix 3). Third, an overview is given of which implementations have proven to be an effective *addition* in an intervention. Finally, the findings of studies that *compared* designs and implementations of strategies are provided.

### Persuasive Strategy: Monitoring

Monitoring involves keeping track of your behavior or behavior outcomes [9]. Traditionally, users had to actively track their own PA behaviors (eg, by means of questionnaires and self-logging); however, nowadays, PA tracking can also be performed passively by using mobile devices, without posing a burden to the user. Almost all of the included studies used monitoring of behavior (28 studies and 82 intervention arms), apart from [54].

#### Design and Technical Implementations

With regard to the technical implementation, 7 different delivery systems were identified that enabled monitoring (for instance, mobile apps [n=46] and SMS functions [n=4]). Most interventions used a combination of 2 systems (mean 1.68). The elements that were used were either related to active self-monitoring (ie, data entry field [n=42]) or passive recordings of behaviors (n=77; eg, accelerometer [n=77], GPS [n=2]). Although most studies used passive tracking, users often had to track their behavior themselves as well. With respect to the design characteristics, many different behavior types were monitored (n=8) and 4 specific monitoring characteristics were identified (such as the option to correct automatically logged data). More details on the various implementations and design characteristics can be found in Multimedia Appendix 3.

#### Effect of Including Monitoring to the Intervention

None of the included studies examined whether adding monitoring to the intervention increased the effectiveness of the intervention.

#### Comparison of Designs and Technical Implementation of Monitoring

One study compared self-logging alone with self-logging in combination with automatic tracking (ie, wearing a second tracking device) regarding its effect on PA. No difference was found in the effectiveness of the 2 technical implementations [41].

### Persuasive Strategy: Goal Setting

Goal setting is a strategy in which the individual either sets a goal or gets a goal assigned. CALO-RE distinguishes between unspecific behavioral goals, behavioral outcome goals, and action plans [9] (also referred to as implementation intentions [65]). It is a commonly used strategy [18], as reflected by the presence of the strategy in most of the included papers (n=26). Not all studies described goal setting in sufficient detail [41,42,61], and these were, therefore, excluded from this part of the analysis. The results of the remaining 23 studies (66 intervention arms) are listed below.

#### Design and Technical Implementations

Regarding the technical implementation, 8 different delivery systems were used to deliver goal setting, of which the researchers themselves were the most common deliverers (n=27). Frequently used elements were reports (n=19) and textual notifications (n=21). Mostly, the goal is only set at the offset of the intervention (n=60), but some systems also changed the goal on a daily (n=3) or weekly (n=6) basis. We found a great variety in the design principles for goal setting; in total, 29 different goal types were identified in the 66 intervention arms. The goal types differed for goal difficulty (eg, 7000 or 10,000 steps) and targeted behavior (eg, step count or floor count; Multimedia Appendix 3). Most of the goals were assigned to the user (n=44); however, in some interventions, the user was instructed to set her own goal (n=16) or the user could choose a goal from a list of suggestions (n=9). Furthermore, 12 specific design characteristics of goal setting were identified, such as tailoring by the system (n=20) and using metaphoric goals (eg, Climb the Eiffel Tower [39]; n=6).

#### Effect of Including Goal Setting to the Intervention

**Figure 1:**
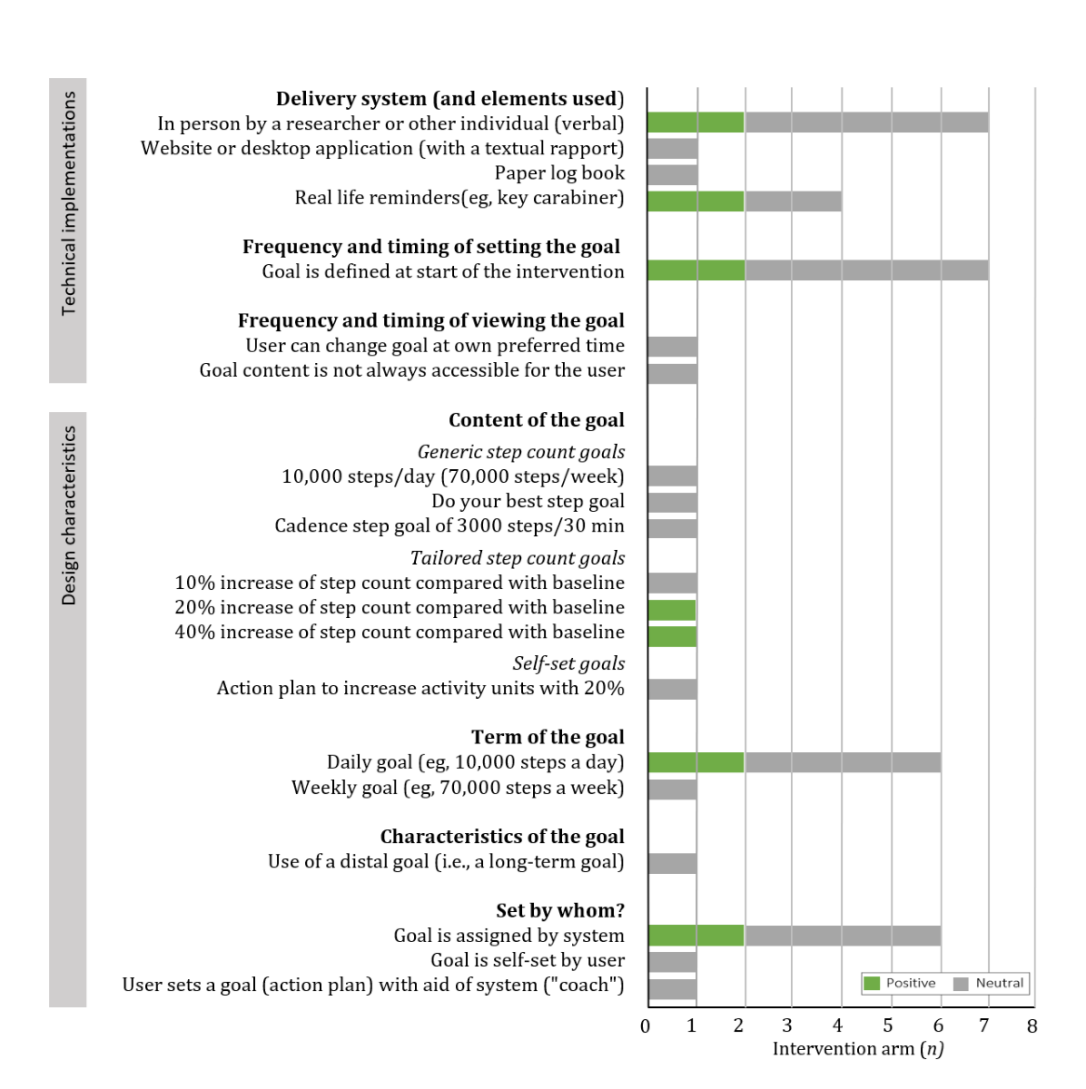
The effectiveness of the technical implementations and design characteristics of goal setting compared with receiving no goals in 7 intervention arms (3 different studies).

In total, 3 studies (7 arms) examined whether including goal setting improved the effectiveness of the intervention. In 2 of the 7 arms, goal setting appeared to be an effective addition [48,50], whereas in the remaining 5, no effect of including goal setting was found [48,50,58]. As can be seen in Figure 1, there is no clear trend in the data regarding effective technical implementations. For the design characteristics of goal setting, it appears that *tailored goals* [50] are generally effective, whereas generic goals do not seem to increase PA behavior [48,58]. However, tailored easy goals (eg, 10% increase compared with baseline) did not result in more PA compared with a group without goal setting [50] (notably, this study lasted for only 1 week). Self-set moderate-to-vigorous PA (MVPA) goals, even with a coaching system for developing action plans, did not result in long-term (48 weeks) PA change compared with a group without goal setting [58]. Thus, these results suggest that some operationalizations of goals increase the effectiveness of the intervention but not all.

#### Comparison of Designs and Technical Implementations of Goal Setting

With 8 studies comparing design characteristics of goal setting [36,37,43,46,48,50,55,57,62], goal setting is one of the most extensively examined strategies. However, no studies have compared various technical implementations of goal setting. The results demonstrate that an effective design for long-term behavior change (4 months) is the use of automatically adaptive and tailored goals compared with a 10,000 steps per day goal [36,62]. Interestingly, a static goal seems to be more effective at the initiation phase of the intervention [36]. A second efficient design is the use of more *difficult* (eg, 40% increase compared with the baseline level) tailored step goals compared with *easier* (eg, 10% increase) tailored step goals [37,50]. However, there is some discrepancy in the right difficulty level; one study found that a step count increase of 20% and 40% of steps was better than a 10% increase [50], whereas another study found that only a 100% increase was better than a 10% increase [37].

A third design that seems effective is using a list of *context-aware* activity goals compared with a list of general not location-specific goals [55]. The context-aware goals were based on previously logged activities and frequent locations of the user to generate location-based goals that were tailored to the individual’s previous behaviors. Finally, the effectiveness of different goal characteristics was examined in one study [46]. Although the differences between the goals did not reach significance, there was a trend demonstrating that participants performed most steps per week if they chose the recommended step goal (mean 42.195), followed by the metaphoric goal (mean 35.462), whereas the self-set goal resulted in the least number of steps (mean 31.774). In contrast, most individuals choose to set a manual goal, which suggests that they prefer this goal setting type.

### Persuasive Strategy: Reminders for PA

Reminders are cues, prompts, or triggers that *push* the user to perform a certain behavior, such as a notification or an email. Only reminders that were used to remind the user to engage in PA were considered. Reminders intended to remind the user to set a goal or to wear the device were not taken into account [40,50,59]. Reminders are used relatively frequently, almost half of the included studies incorporated this strategy (n=13, implemented in 34 intervention arms).

#### Design and Technical Implementations

Regarding technical implementations, 6 different delivery systems were identified (eg, mobile apps [n=13] and email [n=4]) and 6 different delivery elements, such as text notifications (n=24) and visualizations (n=5). The most notable differences in the technical implementations concern the frequency and the timing in which reminders were provided to the users. For instance, in some studies, the user received more than 15 messages per day [42], whereas in other interventions, the user received only one message a week [43] (Multimedia Appendix 3). In contrast to the other investigated strategies, the design characteristics of reminders were often not described in detail. For instance, the framing [24,66], tailoring [23], and size of the messages were seldom reported. Moreover, in some studies, the content of the reminder was not described (n=4).

#### Effect of Including Reminders to the Intervention

**Figure 2:**
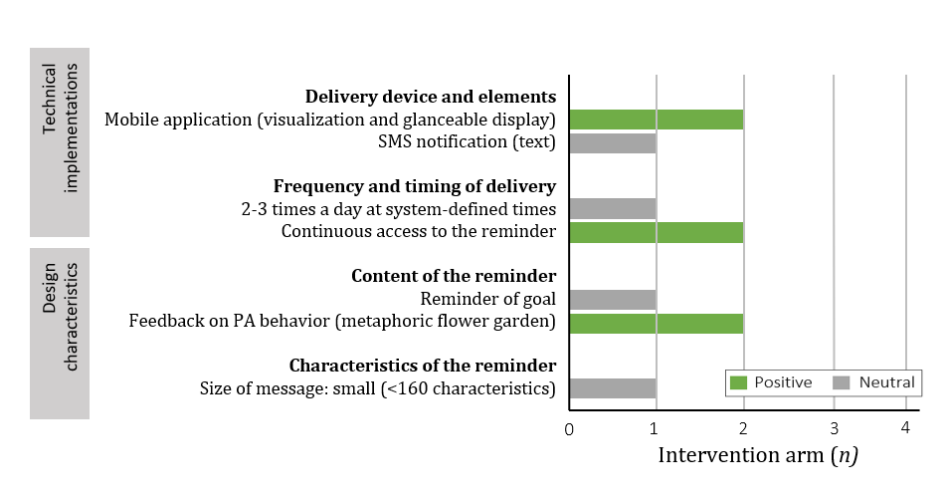
The effectiveness of the technical implementations and design characteristics of reminders compared with receiving no reminders in 3 intervention arms (2 different studies). PA: physical activity.

Only 2 studies (2 intervention arms) examined the effectiveness of receiving a reminder compared with not receiving a reminder, of which one found a positive effect [41] and one found no effect [61]. The design and implementation of the reminders used in these studies and its effectiveness on PA promotion are shown in Figure 2. A study that found a positive effect used a glanceable display, which is a constant reminder that resides on the background of the phone while simultaneously providing the user with information on his or her activity level [41]. The second study demonstrated that 3 SMS notifications a day reminding the user of his or her goal were not effective for increasing PA, at least, not for longer than 1 week [61]. At the end of the intervention, various participants reported that they stopped reading the messages, as the messages were impersonal and automated.

#### Comparison of Designs and Technical Implementations of Reminders

One study examined the effect of the timing of reminders (ie, technical implementation) on the effectiveness of the intervention [42]. The study results were conflicting. On the one hand, individuals increased their steps more if they received reminders at random times (about 10 a day) compared with receiving context-aware reminders (eg, just after eating or after prolonged sedentary time; no significance values were reported) [42]. On the other hand, the participants liked and accepted the context-aware reminders better than random reminders. The authors argue that these conflicting findings might be the result of the short duration of the study and a few participants (n=19), which increases the likelihood of factors (weather and busy calendar) influencing the results. A second study examined whether the content of reminders influences its effectiveness (ie, the design characteristics) [54]. No differences in step count were found between receiving reminders of the participants’ action plans or of their general goal.

### Persuasive Strategy: Rewards

Rewards are reinforcers of behavior that can be given for attempts to reach a goal and for reaching the goal [9]. In line with the CALO-RE taxonomy [9], only rewards that incentivize performing PA behaviors were considered and not rewards that incentivize study participation. A total of 12 studies (29 different intervention arms) met these criteria.

#### Design and Technical Implementations

Regarding technical implementations, 6 different delivery systems were used, such as websites (n=14). Furthermore, 7 different elements were identified, the most frequent element being visualizations (n=13). Most participants received rewards either immediately after they achieved their goal (n=14) or with a short delay (n=10). In a few studies, participants were rewarded for performing PA (eg, each step taken) and not necessarily for reaching their goal (n=6). The design characteristics varied greatly between interventions (Multimedia Appendix 3). For instance, we identified 16 different reward contents (eg, points, US $1 per achieved goal [n=2]), 9 different behaviors that were rewarded (eg, achieving 7000 steps per day [n=8]) and 7 reward characteristics (eg, receiving the reward depended on somebody else [n=8]).

#### Effect of Including Rewards to the Intervention

**Figure 3:**
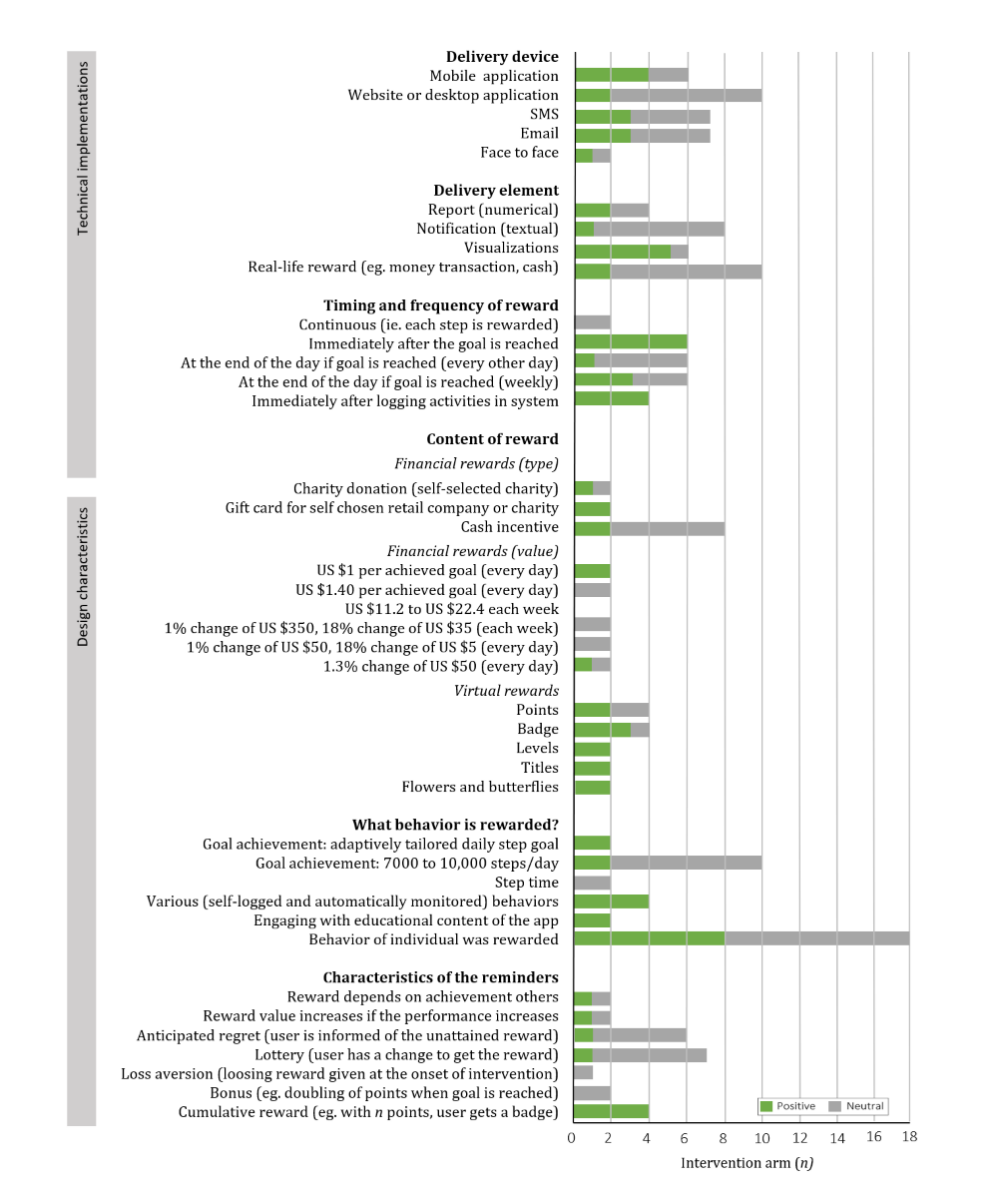
The effectiveness of the technical implementations and design characteristics of rewards compared with receiving no rewards in 18 intervention arms (8 different studies). PA: physical activity.

Of the identified studies (18 intervention arms), 8 examined whether including rewards increases the effectiveness of the intervention [36,41,43,44,51-53,63]. Of the 18 arms, 8 arms demonstrated that adding a reward resulted in more PA than the same intervention without rewards [36,41,43,51], whereas the remaining 10 did not have a significant effect on PA [43,51-53,63]. Some technical implementations of rewards appear to be effective additions to interventions (Figure 3), such as the use of visualizations of the rewards [41,43] and receiving the reward immediately after the goal is attained [36,41,43]. Implementations that do not seem to result in more PA are rewarding each step (ie, efforts toward reaching a goal) [63]. Furthermore, interventions with the design characteristic of *cumulative rewards* (eg, with enough points the user receives a badge) appear to be effective in motivating individuals to engage in PA [36,41,43,51]. Taken together, it seems that some operationalizations of rewards are effective additions to an intervention, whereas others are not.

#### Comparison of Designs and Technical Implementations of Rewards

A total of 3 studies compared different design characteristics of (financial) rewards [44,51,52]. Finkelstein et al [44] demonstrated that adding a cash reward on top of virtual rewards (from Fitbit) increases step count, and they also showed that cash rewards are equally effective in changing the stepping behavior as charity rewards. Notably, the MVPA minutes (main outcome of the study) was higher in the group that received a cash reward [44]. Furthermore, when the financial reward was no longer offered (after 12 weeks), the step count declined. Other studies found no significant differences between rewarding the behavior of the individual, the behavior of a team, or a combination of both [51] and between receiving a financial reward, a loss aversion reward, or a lottery-based reward [52].

### Persuasive Strategy: Sharing

Sharing is a social strategy in which users can actively offer and receive social support from others. It can be provided digitally (eg, sharing on Facebook) or in a real-life setting (eg, group meetings [48]). In total, 9 studies (15 intervention arms) were identified that included this strategy in their intervention.

#### Design and Technical Implementations

Regarding the technical implementation of sharing, 4 different delivery systems were used: mobile apps (n=8) and face-to-face delivery (n=3). In total, 8 different delivery elements were identified, such as reports and messaging functions (Multimedia Appendix 3). Various design characteristics were also identified. For instance, several content types could be shared, such as PA data (n=5) and competition results (n=2). In addition, the relationships between individuals who shared information with each other differed between studies. Sometimes, the users shared their information with strangers (n=5), whereas in other interventions, individuals shared their information with acquaintances (n=4) or acquaintances and strangers (n=2).

#### Effect of Including Sharing to the Intervention

**Figure 4:**
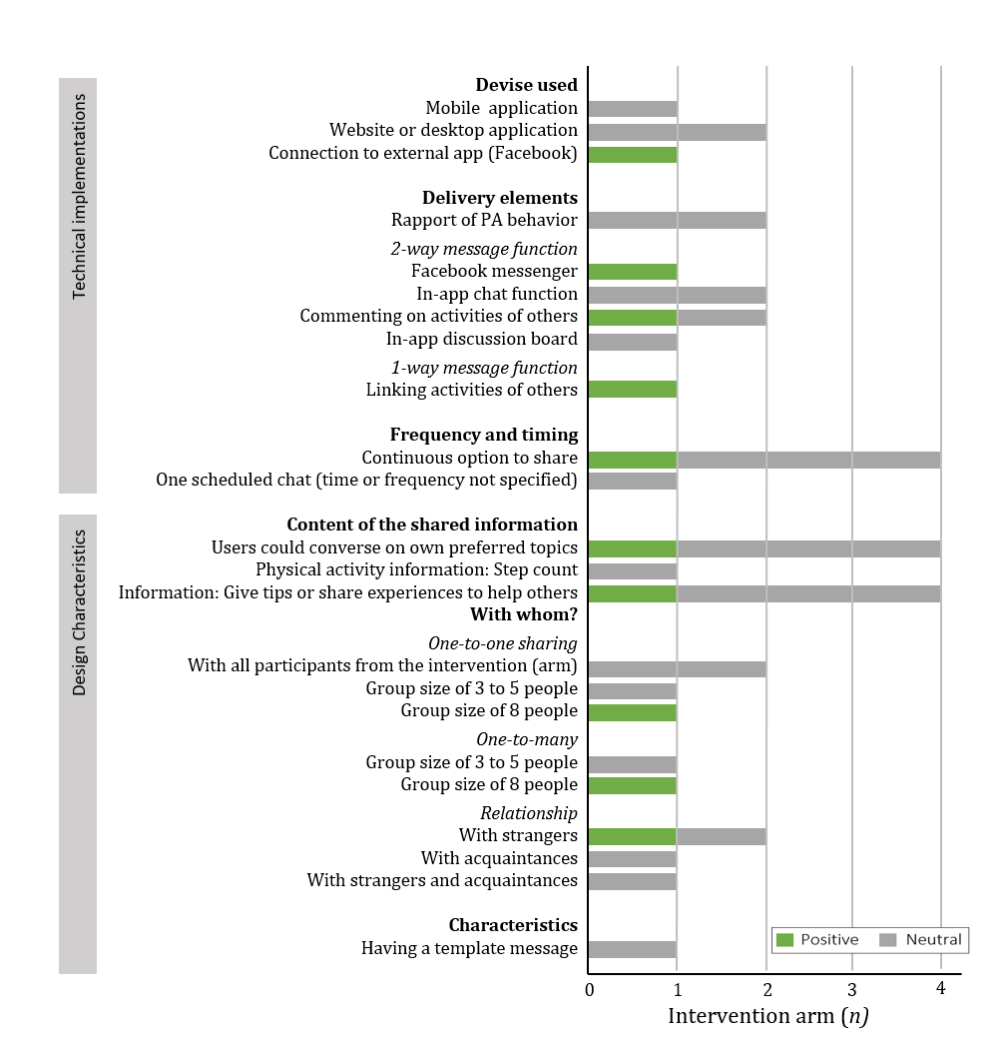
The effectiveness of the technical implementations and design characteristics of sharing compared with receiving no sharing function in 4 intervention arms (4 different studies). PA: physical activity.

In total, 4 studies (4 intervention arms) examined whether adding sharing strategies to the intervention increased its effectiveness [40,49,56,60]. One study found a positive effect [56], and the other 3 found no effect of sharing on PA [40,49,60] (Figure 4). The interventions differed in devices that were used for sharing. In a study with positive results, individuals (frequent Facebook users) used Facebook [56], whereas studies with no effect used websites or a mobile app (specifically developed for the particular study) [40,49,60]. A Facebook-delivered intervention is likely to be effective because it is well integrated into the individual’s life. In contrast, using an additional intervention website might pose a barrier for the user, which can explain why they were neither effective nor frequently used [49,60].

#### Comparison of Designs and Technical Implementations of Sharing

As none of the included studies compared different designs or technical implementations of sharing to examine which implementation or design is more effective in increasing PA, no conclusions can be drawn on effective operationalization of sharing to increase PA behavior.

### Persuasive Strategy: Social Comparison

A second social strategy is facilitating *social comparison* [9], which includes competition, collaboration, and social norm information. In total, 9 of the included studies enabled social comparisons (17 intervention arms and 11 did not include social comparison).

#### Design and Technical Implementations

A total of 4 different delivery systems were used, of which mobile apps were most frequently used (n=8). To enable social comparison, almost all studies used reports (n=16) and some included visualizations (n=3; Multimedia Appendix 3). In most studies, the user could view the comparison at all times (n=10); however, sometimes the participant received a comparison message at fixed times (for instance, once a week [n=4]). Regarding the design characteristics, different types of comparisons were identified, including competition (ie, individual vs individual [n=5] and team vs team [n=2]), collaboration between individuals (n=4), and various forms (n=3) of social norm information (eg, the individual’s average step count compared with the step count of all users) [45,47,59,60]. Furthermore, 3 different PA behaviors were compared across the interventions and 7 different social compositions (eg, between 2 friends or with a group of strangers) were identified.

#### Effect of Including Social Comparison to the Intervention

**Figure 5:**
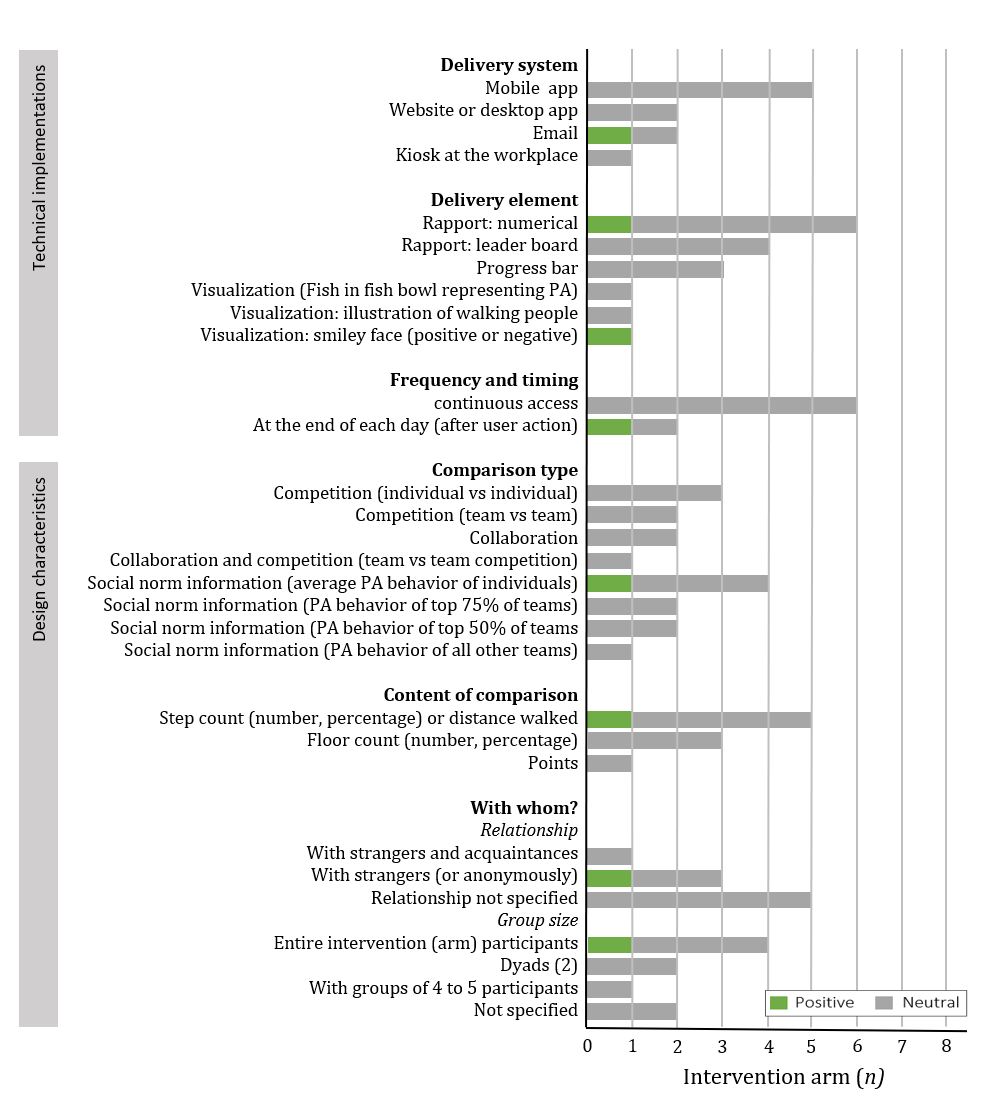
The effectiveness of the technical implementations and design characteristics of social comparison compared with receiving no comparison in 9 intervention arms (6 different studies). PA: physical activity.

In total, 6 studies (8 intervention arms) examined whether adding a comparison with an intervention increased the effectiveness of the intervention [39,45,47,59,63,64]. One study (1 intervention arm) found that including social strategies resulted in more PA [59], although it did not significantly increase PA behavior in the remaining 7 arms (Figure 5) [39,45,47,63,64]. The effective implementation involved weekly reports (emails) to inform the user if he or she performed more steps than the average study participants. If the user did, the email contained a positive smiley face. Otherwise, a negative smiley face was placed in the report [59].

#### Comparison of Designs and Technical Implementations of Social Comparison

A total of 2 studies were identified that compared the effect of different design characteristics of social comparisons on PA behavior [39,59]; however, no study examined the technical implementations. One of these studies found that competition was less effective than collaboration or a combination of collaboration and competition (in the short term, ie, 2 weeks [39]). In a second study, the step count of the individual’s team (4 individuals) was compared with either the top 25% or the top 50% of the other teams. No significant differences were found between the groups [59], suggesting that this characteristic does not influence the effectiveness of the intervention.

## Discussion

### Principal Findings

The main objectives of this study were to examine (1) the variation in the technical implementations and design characteristics of common persuasive strategies and (2) if previous mHealth studies examined the influence of the different operationalizations of these persuasive strategies on the effectiveness of the intervention to persuade the user to engage in PA. Similar to previous work on feedback [22], we found that the technical implementations and design characteristics of the examined persuasive strategies vary greatly between studies. For instance, 29 different goal types were identified in the 29 included studies. The goals differed in terms of behavior type (eg, step count, time walked) and difficulty (eg, 10,000 steps per day, 7000 steps per day).

Only a few of the identified implementations and designs were thoroughly examined for effectiveness, especially regarding the technical implementation. Interestingly, the studies that examined this showed that some implementations and designs are more effective in increasing PA compared with others. By performing an in-depth analysis of these studies, this review provides important insights into which implementation types and designs are promising for PA mHealth interventions, as listed below. Furthermore, this study highlights that more research on effective technical implementations and design characteristics of persuasive strategies is essential. 

### Monitoring

Monitoring was not only the most frequently used strategy in the selected studies but also the least investigated one regarding implementation types and designs. Only one study compared technical implementations, which found no significant difference regarding intervention effectiveness between automatic tracking alone, or both automatic tracking and (active) self-monitoring [41]. Thus, although self-monitoring is arguably more demanding for the user, these results suggest that this additional effort does not influence the effectiveness of the intervention. A possible explanation is that individuals perceive wearing a second device as inconvenient or not pretty (eg, when wearing a dress) [40], which counterbalances the positive effects of the ease of use of automatic tracking. More research is needed to better understand how monitoring can be implemented to successfully support PA behavior.

### Goal Setting

The results suggest that the operationalization of goal setting influences the effectiveness of the intervention to increase PA behavior. In line with the Goal Setting Theory [34], this review demonstrates that goals that are tailored to the physical capabilities of the user and/or the context of the user are likely more effective in increasing PA than generic goals [36,37,50,55,62]. Next, challenging (but doable) goals appear to be more effective than easy goals. However, there is no consensus on how challenging the goal should be. Is a small increase of baseline steps a difficult enough goal (eg, 20% increase [50]) or is a large increase required (eg, a 100% increase [37])?

In contrast to the Goal Setting Theory [34] and other theories, such as the Self-Determination Theory (SDT) [67], positing that self-set goals are likely to be more effective, the results of this review indicate that adding self-set goals to an intervention does not increase effectiveness [58] and that goals set by the system might be more promising than self-set goals in increasing PA behavior [68]. However, people will likely choose to set their own goal when offered the choice [68]. Therefore, there might be a discrepancy between what the user wants and what is most effective. Whether a combination of both self-set and assigned goals is effective should be determined in future studies. Future efforts are also needed to explore the technical implementations of gamification, as none of the selected studies examined this.

### Reminders

One of the included studies in this study demonstrated that a promising approach to implement reminders is the use of glanceable displays, which is a constant but gentle reminder that resides at the background of the device [41]. However, as most implementation types of reminders were not compared with each other, it is unknown if certain designs of glanceable displays are more attractive and effective than others (eg, *flowers or a robot army* [41]) or if other types of reminders (such as text messages) are equally effective as glanceable displays.

With respect to the design characteristics of reminder text messages, tailoring the timing of the messages might improve the intervention [4,33,69]. One study examined this and found that optimally timed messages were perceived as more useful than randomly timed messages, although it did not result in more PA [42]. Next, regarding the content of the reminders, the results of this study suggest that *generic* reminders do not result in more PA compared with receiving no messages [61]. People perceive these messages as impersonal, *boring* [61] and stop reading them [70,71]. Tailored message content is likely more appropriate [23], as demonstrated in similar research domains [72]. However, none of the papers selected for this study examined this. Taken together, future studies should be conducted to better understand the effectiveness of tailoring (of both the content and the timing) of messages to increase PA behavior.

### Rewards

This study suggests that the design and implementation of rewards are important factors that motivate individuals to engage in PA. For instance, the results suggest that rewards are effective if they are cumulative (eg, with enough points, the participant receives a badge). Furthermore, receiving an immediate reward (eg, immediately after attaining the goal) is likely effective, whereas receiving delayed rewards seems less effective. This is in line with previous research and behavioral economics theory [35], which explain that immediate rewards are perceived as more valuable than rewards given in the future [73,74].

Although several studies have examined effective reward types (eg, comparing monetary rewards with charity donations), multiple important questions remain unanswered. For instance, it is neither known whether the value of the financial reward influences its effectiveness, nor is it known if certain types of virtual rewards (eg, badges, points, levels) are more effective than others. The use of rewards is sometimes criticized, as they mainly promote the extrinsic motivation to perform PA [75,76] and can inhibit the intrinsic motivation associated with lasting behavior change. Consequently, users likely stop performing PA after the reward is removed, as demonstrated in several of the selected studies of this study [44,51,52]. Therefore, it is important to gain a better understanding of how to design rewards that foster feelings of intrinsic motivation [77].

### Sharing

Sharing and giving social support are argued to foster feelings of relatedness to others, which is an important determinant of intrinsic motivation to perform PA according to the SDT [67,75]. However, of the 4 studies that examined the effect of adding sharing to the intervention, only 1 study found that having access to a sharing function (with Facebook) significantly increased the step count [56]. A possible explanation for the ineffectiveness of the other 3 implementations of sharing is that they used a delivery system that is not well integrated in people’s lives and are, therefore, not frequently used [40,49,60]. However, as no study has directly compared the effectiveness of different delivery systems, future research should examine whether the delivery system of social sharing indeed influences the effectiveness of the intervention. Other technical implementation or design characteristics also require future investigation, such as with whom the data are shared or the type of data that are shared (such as tips from peers or the performed PA behavior).

### Social Comparison

In contrast to previous work on PA (mHealth) interventions [7,14], we found that most interventions that included social comparisons did not result in significantly more PA compared with the same intervention without social comparison. There may be various reasons for this, such as the relatively small sample sizes used in the studies [38,47] because the strategy was not optimally operationalized or it could be that the strategy itself is not effective. As only 2 studies compared different design characteristics and no study examined the technical implementation of social comparisons, knowledge on effective operationalizations remains to be limited. However, it does seem that collaboration enhances participation in PA above competition (in line with previous research [38,78]). Notably, it might not be competition itself but the overemphasis on winning that can be counterproductive [78]. In general, it is thought that competition can increase PA when everybody has a reasonable chance to win [79], as also indicated in the exit interviews of Chen and Pu [38].

### Strengths, Limitations, and Recommendations

The important strengths of this study are the in-depth systematic analysis of multiple persuasive strategies by using a framework and the exploration of both the technical implementation and design characteristics of the interventions. By doing so, we went beyond examining which persuasive strategies are effective by also examining how these strategies can be effectively implemented and designed. Furthermore, by only including studies that evaluated the isolated effect of the selected persuasive strategies, an overview of implementations and designs could be provided that have been shown to be effective.

However, this study has some limitations. First, inherent to performing scoping reviews, we did not consider the quality of the included studies, which could have biased the results. In addition, as no systematic search was performed, important literature could have been overlooked. To minimize this risk, we performed cross-reference checks of previous (recent) reviews and snowball searches. In light of these limitations, the results of this study should be treated with caution. Second, no corrections for other factors that can influence the effectiveness of the intervention, such as the study duration [36], the geographical location of the study [30], or user friendliness of the intervention [27]. To illustrate, it is likely that the same intervention is not equally effective in different contexts (such as in different countries or seasons). Although we recognize the importance of these factors, it was beyond the scope of this paper to examine this. A third limitation is that the influence of other persuasive strategies of the intervention was not examined. For instance, it is possible that the investigated strategy did not increase PA behavior, even though its operationalization was good, as the other included persuasive strategies of the intervention were not set up properly.

Furthermore, the data extraction was limited to the amount of details in which the researchers described their intervention. For instance, most papers did not describe how they developed the reminder system (eg, whether they used a pool of messages or framed the messages positively or negatively). Therefore, we call on future studies to report the implementations and designs in more detail. For instance, authors can include screenshots of the apps or websites [43], videos of the app, and/or a user manual. To document mHealth interventions, researchers can use the framework provided in Multimedia Appendix 3 and, for instance, the work of Schembre et al [22], Elaheebocus et al [25], and/or Hoffman et al [80].

We realize that the number of implementation types is so diverse that it would cost considerable time and resources to investigate the individual design characteristics and technical implementations of the strategies. A promising approach to reduce this burden on researchers is to build a database in which mHealth PA interventions are described at a granular level, covering both the implementation characteristics of the included persuasive strategies and the study characteristics (as possible confounders). Advanced statistical testing (eg, Meta-Cart analyses [81]) and machine learning techniques [82] allow identifying which implementation characteristics seem effective for which target group.

### Conclusions

Mobile exercise interventions have the potential to increase PA behaviors of individuals [1,6,7]. However, there is a limited understanding of how to effectively develop exercise interventions and its components (ie, their persuasive strategies). To increase this understanding, it is important to examine the operationalization of persuasive strategies and to evaluate its impact on the effectiveness of the intervention. The results of this study highlight the great variation in which monitoring, goal setting, reminders, rewards, sharing, and social comparison are being operationalized. Moreover, the findings of this study suggest that how a conceptual persuasive strategy is being translated into a practical delivery form can influence the effectiveness of the PA intervention. Thus, the operationalization of strategies in mHealth interventions should be critically considered when developing such interventions and before drawing conclusions on the effectiveness of the strategy as a whole. To advance the research field, future research should go beyond evaluating *which* persuasive strategies are effective by also examining *how* these strategies can be effectively implemented and designed. 

## Appendix

Multimedia Appendix 1 The PRISMA ScR (Preferred Reporting Items for Systematic Reviews and Meta-Analyses Extension for Scoping Reviews) checklist.

Multimedia Appendix 2 The data chart used for extracting information from the selected papers consisting of (1) general study characteristics, (2) technical implementations and design characteristics of persuasive strategies, and (3) study results.

Multimedia Appendix 3 The characteristics of the included studies and the design characteristics and technical implementations of monitoring, goal setting, reminders, rewards, social support, and social comparisons.

## Abbreviations

BIT: Behavioral Intervention Technology
mHealth: mobile health
MVPA: moderate-to-vigorous physical activity
PA: physical activity
SDT: Self-Determination Theory
