# Supplementary material for: Unraveling Mobile Health Exercise Interventions for Adults: Scoping Review on the Implementations and Designs of Persuasive Strategies
Source: JMIR Mhealth Uhealth. 2021 Jan 18;9(1):e16282. doi: 10.2196/16282 (PMC7850911; doi:10.2196/16282)
Supplement: Multimedia Appendix 2 [file mhealth_v9i1e16282_app2.docx]

Multimedia Appendix 2. Data chart consisting of (1) general study characteristics, (2) technical implementations and design characteristics of persuasive strategies and (3) study results.

| **Extracted variable** | | | **Description** |
| --- | --- | --- | --- |
|  | | | |
| *General study characteristics^a^* | | |  |
|  | Author | | The authors of the paper |
|  | Year of publication | | The year in which the study was published |
|  | Title | | Title of the paper |
|  | Keywords | | The keywords used by the authors. |
|  | Theoretical embedding | | The behavioral theories that were mentioned by the authors in the paper as a rationale for the intervention (strategies). |
|  | Journal | | The journal which published the paper |
|  | Intervention goal | | The goal described by the authors regarding the study. |
|  | Intervention type | | The type of experimental study design used (e.g. RCT, pre-test- post-test) |
|  | Experimental design | | The experimental design of the study, divided in randomized controlled trials (1), factorial designs (2) or other designs (3) |
|  | Control group | | The study included a control group (intervention group that did not receive the intervention) (1) or no control group (0) |
|  | Baseline comparison | | The study included a baseline measurement (1) or no baseline measurement (0) |
|  | Baseline measurement type | | If the study included a baseline measurement, the type of baseline measurement is described. |
|  | Intervention duration | | The duration of the intervention period (not including baseline or follow-up) |
|  | Follow up measurement | | The study included a follow up measurement (does not include exit questionnaires)(1) or no follow up measurement (0) |
|  | Follow up measurement characteristics | | If the study included a follow up measurement, the type of baseline measurement is described. |
|  | Target group | | The target group of the interventions |
|  | In/exclusion criteria | | The in/exclusion criteria of the participants |
|  | Number of participants | | The number of participants that finished the study |
|  | Country/city | | The country (and region or city, if specified) where the study was performed. |
|  | Setting | | If the study was performed in a specific setting (e.g. at the workplace) it was described. |
|  | Controlling for setting | | If the study controlled for setting or factors of the physical environmental, it was described. |
|  | Season | | If the study controlled for the season or weather type in which the study was performed |
|  | Controlling for season or weather related factors | | If the study controlled for seasonal or weather related factors it was described. |
| *Mobile intervention characteristics* | | |  |
|  | App goal^a^ | | The type of behavior the app intends to change |
|  | Persuasive strategy (BIT: Conceptual “how”)^b^ | | The strategies that were used in the intervention (limited to the strategies which are investigated in the current review, i.e. monitoring, goal setting, rewards, reminders, social strategies) |
|  | *Technical characteristics ^c^* | |  |
|  |  | BIT: Device used | The device of delivery of the persuasive strategy (e.g. mobile phone, email, website) |
|  |  | BIT: Delivery elements | The mode of delivery of the persuasive strategy (e.g. text message, visualizations, ) |
|  |  | BIT: Workflow | The conditions under which the user received the persuasive strategy (e.g. time, frequency, decision rules) |
|  | *Design characteristics^c^* | |  |
|  |  | BIT: Characteristics of the element | What were the characteristics of the intervention (e.g. framing of the message, difficulty of the goal, sharing with how many individuals). |
| *Study results* | | |  |
|  | Compared to baseline^b^ | | The effect of the intervention compared to baseline. |
|  | Compared to control^b^ | | The effect of the intervention compared to a control group. This was only reported when the control group received the same intervention, apart from the persuasive strategy in question |
|  | Compared to other implementation^b^ | | The effect of the intervention compared to a another intervention arm. This was only reported when the other intervention arm received the same persuasive strategy, but implemented or designed differently. |

^a^ data was extracted for the study as a whole

^b^ data was extracted for each study arm

^c^ data was extracted for each persuasive strategy
